# The new bone formation in human maxillary sinuses using two bone substitutes with different resorption types associated or not with autogenous bone graft: a comparative histomorphometric, immunohistochemical and randomized clinical study

**DOI:** 10.1590/1678-7757-2020-0568

**Published:** 2020-12-18

**Authors:** Rodrigo dos Santos PEREIRA, João Paulo BONARDI, Felippe RicardoFrossard OUVERNEY, Annelise Backer CAMPOS, Geraldo Luiz GRIZA, Roberta OKAMOTO, Eduardo HOCHULI-VIEIRA

**Affiliations:** 1 Universidade do Grande Rio Centro Universitário Serra dos Órgãos Brasil Universidade do Grande Rio (UNIGRANRIO); Centro Universitário Serra dos Órgãos (UNIFESO).; 2 Centro Universitário Serra dos Órgãos Brasil Centro Universitário Serra dos Órgãos (UNIFESO).; 3 Universidade Estadual Paulista Júlio de Mesquita Filho Brasil Universidade Estadual Paulista Júlio de Mesquita Filho (UNESP).; 4 Universidade Estadual do Oeste do Paraná Brasil Universidade Estadual do Oeste do Paraná (UNIOESTE).

**Keywords:** Biomaterials, Bone substitutes, Sinus floor augmentation, Tissue physiology, Bone regeneration, Xenograft, Bioactive glass

## Abstract

**Objective:**

The aim of this study is to evaluate the new bone and connective tissue formation and the biomaterial remaining after maxillary sinus bone augmentation using 5 different bone substitutes. The osteocalcin immunolabeling was performed to demonstrate their calcification and the possibility of receiving dental implants.

**Methodology:**

40 patients underwent maxillary sinus bone augmentation and were divided in 5 groups: Group 1 with 8 maxillary sinuses were grafted with autogenous bone graft (AB); Group 2 with 8 maxillary sinuses grafted with bioactive glass (BG); Group 3 with 8 maxillary sinuses grafted with bioactive glass added to autogenous bone graft (BG + AB) 1:1; Group 4 with 8 maxillary sinuses grafted with Bio-Oss (BO) and Group 5 with 8 maxillary sinuses grafted with Bio-Oss added to autogenous bone graft (BO + AB) 1:1.

**Results:**

In group AB, 37.8% of bone was formed in the pristine bone region, 38.1% in the intermediate and 44.5% in the apical region. In group BG, 43.6% was formed in the pristine bone, 37% in the intermediate and 49.3% in the apical region. In group BG + AB 1:1, 39.0% was formed in the pristine bone region, 34.8% in the intermediate and 36.8% in apical region. In group BO, 33.4% was formed in the pristine bone, 32.5% in the intermediate and 34.3% in the apical region. In group BO + AB 1:1, 32.8% was formed in the pristine bone, 36.1% in intermediate and 27.8% in the apical regions. The immunolabeling for osteocalcin showed an intensive staining for all groups, which could demonstrate the calcification of the bone formed.

**Conclusion:**

This study showed that the groups evaluated formed a suitable lamellar bone in the maxillary sinus reconstruction after six months of bone healing, thus being indicated to receive dental implants.

## Introduction

Physiological phenomena such as bone resorption and maxillary sinus pneumatization occur when the posterior maxillary teeth are lost.^1^ Since oral rehabilitation using dental implants is impossible at this point, bone reconstruction is the procedure of choice.

Different surgical techniques that depend on the residual bone on the maxillary sinus floor have been developed.^2^ The posterior maxillary reconstruction is then considered a predictable and reliable procedure. Since its creation by Boyne and James, different bone substitutes have been used as autogenous bone grafts, xenografts and allografts, among others.^3-5^

However, an ideal bone substitute is yet to be described in the literature.^6^ Among the numerous biomaterials created and researched over the years, the autogenous bone graft (AB) is considered the “gold standard” due to its specific properties, namely osteoconduction, osteoinduction, and osteogenesis.

Bio-Oss^®^ (BO) (Geistlich Biomaterials, Wolhusen, LU, Switzerland) is the most used bone substitute, consisting of a deproteinized bovine bone with osteoconductive properties that has shown promising results due to its morphology similar to the human bone.^7,8^ Bioactive glass (BG) (Biogran; Biomet, Warsaw, IN, USA) is another biomaterial with promising results, consisting of an osteoconductive ceramic with peculiar properties of graft resorption due to the chemical dissolution.^9,10^

The aim of this study was to compare the new bone formation in human maxillary sinuses grafted with these two bone substitutes (Biogran & Bio-Oss), which have different resorption pathways, using histomorphometric and immunohistochemical analysis, and to determine the effects of their addition to autogenous bone graft (1:1) after 6 months of bone healing.

### Hypothesis

H_0_ – There will be no difference in the new bone formation rates using biomaterials of different resorption pathways.

## Methodology

### Human Subjects

All procedures performed in studies involving human participants were in accordance with the ethical standards of the institutional and national research committee approved with the number 47711015.4.0000.5420 by Plataforma Brasil/CONEP; with the 1964 Helsinki Declaration and its later amendments.

**Figure 1:**
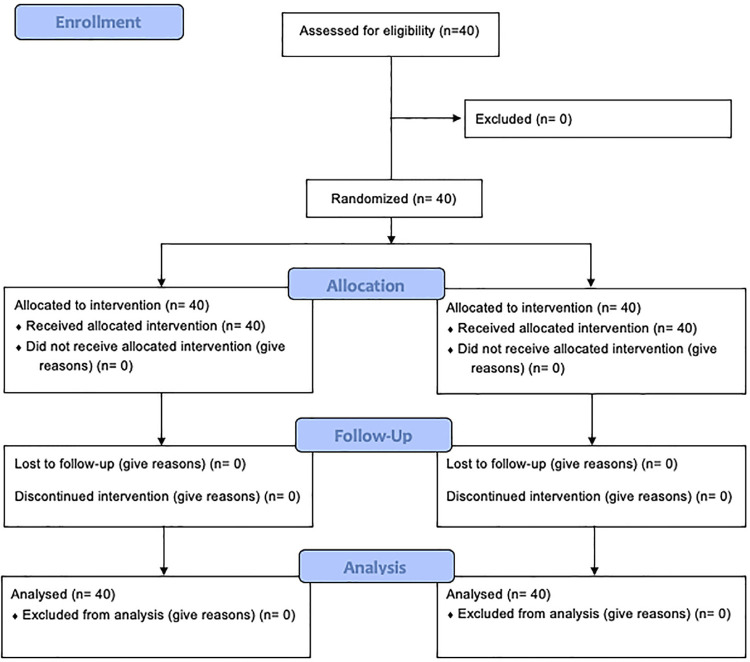
CONSORT diagram of the patient allocation by randomization

Quality assessment was conducted according to the CONSORT Statement’s RCT checklist^11^ (Figure 1).

### Sample size

Based on previous studies, the number of maxillary sinuses to be grafted were determined by performing a power test on the website www.lee.dante.br ^12^. For this test, the difference of the mean was 15.1, with a standard deviation of 9.9. The test was conducted in a 1-tailed hypothesis with a 5% level of significance and a 80% power. The results indicated a minimum of 5 samples for each group.

### Randomization

Drawing lots was performed to randomize and decide the sites to be grafted with each material by a clinical assistant.

### Inclusion & exclusion criteria

All patients were subjected to facial cone beam computed tomography (CBCT) to evaluate the maxillary sinuses, as well as the proximity of mandibular canal and inferior dental roots where the autogenous bone graft were harvested. The inclusion criteria were: patients with maxillary sinuses with less than 5 mm of bone remaining; those who decided to be rehabilitated with dental implants; and those with bone in the mandibular symphysis or in the retromolar region. Exclusion criteria were: patients that reported uncontrolled systemic disease, untreated periodontal disease, or sinus pathologies; smokers; patients without enough bone at the mandibular regions to be harvested for the autogenous bone graft; patients who reported medication-related osteonecrosis, or patients treated for head and neck cancer.

### Group determination

Based on the aforementioned parameters, 40 patients were selected for unilateral maxillary sinus bone augmentation and divided into 5 groups:

Group 1: eight maxillary sinuses grafted with AB as control group;

Group 2: eight maxillary sinuses grafted with BG;

Group 3: eight maxillary sinuses grafted with BG + AB 1:1;

Group 4: eight maxillary sinuses grafted with BO,

Group 5: eight maxillary sinuses grafted with BO + AB 1:1.

### Surgical procedure

Surgical procedures were performed under local anesthesia using lidocaine 2% with 1:100 epinephrine (DFL; Taquara, RJ, Brazil). The autogenous bone graft was harvested from retromolar region by vestibular approach similar to that of mandibular sagittal osteotomy and osteotomized using 701 drill. The harvesting from symphysis were performed by an intraoral anterior vestibular approach, 5 mm below the gingival line extending from canine tooth. The bone was also osteotomized using a 701 drill and removed using a chisel.^9,12,13^ The bone blocks were grounded with a bone crusher (Neodent; Curitiba, PR, Brazil). The maxillary sinuses were treated according to Boyne and James^3^ (1980) using a posterior maxillary approach to expose the lateral wall of maxillary bone followed by a osteotomy using a nº 6 sphere drill to access the Schneiderian membrane and its elevation. The bone grafts were mixed using a syringe in cc to allow the 1:1 proportion. Post-operative pain was treated with 500 mg paracetamol (EMS; São Paulo, SP, Brazil), prescribed four times per day and 500 mg amoxicillin (EMS; São Paulo, SP, Brazil) was prescribed three times per day to reduce the chances of infection.

### Histology & histomorphometric analysis

After 6 months of bone repair, bone biopsies were harvested using a 3.0 mm × 15 mm trephine bur (MK Life; Porto Alegre, RS, Brazil) at the time of dental implants placement and stored in a 10% formalin solution (pH 7) for 48 h. The samples were washed in running water for 24 hours, decalcified for 4 weeks in an EDTA solution changed weekly. Subsequently, they were embedded in paraffin; sectioned and stained with hematoxylin and eosin. Samples were identified and stored following the apical orientation in all sequence during laboratory procedures. The biopsies were evaluated according to Pereira, et al.^12^ (2017) in three regions using a light microscopy with a digital camera attached to capture the images at ×12.5 magnification:

- pristine bone (2 mm above the maxillary sinus floor),
- intermediate,
- apical (2 mm below the membrane)

To exclude the influence of maxillary sinus floor and Schneiderian membrane during the evaluation, a 2 mm distance from upper and lower regions was respected. The height of pristine bone region was measured in the CBCT previously performed to exclude it during the microscope visualization. New bone formation, amount of connective tissue and the remaining biomaterial were analyzed by histometry using a grid of Merz^14^ (1968).

### Immunohistochemical evaluation

The immunohistochemical analysis was performed according to Pereira, et al.^15^ (2017). Primary polyclonal goat antibodies against human Osteocalcin (Santa Cruz Biotechnology; CA, USA; SC18319) were used in immunohistochemical assays to identify calcified tissue.^16^ A biotinylated donkey anti-goat secondary antibody (Jackson Immunoresearch Laboratories, West Grove, PA, USA) coupled to avidin (Vector Laboratories, Burlingame, CA, USA) was used for signal amplification. The binding reaction was detected with diaminobenzidine (Sigma-Aldrich, St Louis, MO, USA). Data analyses were performed using a single-evaluator semi-quantitative approach, with score “0” indicating the absence of staining and scores “1,” “2,” or “3” indicating low, moderate, or intense staining, respectively.

### Statistical analysis

The Kolmogorov-Smirnov test was performed to indicate the parametric or non-parametric distribution of the samples. In case of normal distribution, a comparison among the groups was made using the ANOVA test followed by Tukey’s multiple comparison test. In case of non-parametric distribution, a Kruskal-Wallis test was performed. A priori, p-value<0.05 was considered significant for all tests.

## Results

Forty patients (22 men and 18 women) aged between 32 and 65 years old were subjected to unilateral maxillary sinus bone augmentation using the 5 types of bone grafts purposed in our study.

### Histology & histomorphometric outcomes

The group AB presented predominant lamellar bone formation in all three regions evaluated, with the presence of osteocytes in the bone matrix. The mean for the new bone formed was 37.8%±16.9 in the pristine bone region; 38.1%±21.4 in the intermediate region and 44.5%±18.6 in the apical region. The connective tissue formed presented a mean of 57.6%±16.3 in pristine bone; 58.8%±20.9 in intermediate and 54.0%±16.5 in apical region. The median for the remaining biomaterial was 1.5 in the pristine bone region, 0.5 in the intermediate region and 2.5 in the apical region.

The group BG showed new bone formation, which were in the process of lamellar organization in all three regions. However, in most cases, the presence of lamellar bone organization was observed. We also observed the presence of cellularized connective tissue with remaining bioactive glass particles and osteoblasts in the periphery of the bone matrix. The mean of new bone formed was 43.6%±4.7; 37%±10.9 and 49.3%±13.2 in the pristine bone, intermediate and apical region, respectively. The connective tissue presented a 56.6%±6.5 mean for pristine bone; 63.4%±11.0 for intermediate and 49.3%±13.2 for apical region. The median for the 3 regions evaluated for the present group was 0.

In group BG + AB 1:1, a new bone formation with lamellar organization were observed, mostly showing more woven bone formation presented in the apical region. The connective tissue had cells and osteoblasts in the periphery of the bone matrix. The mean for new bone formed was 39.0%±15.8 in the pristine bone region; 34.8%±14.5 in the intermediate and 36.8%±14.5 in apical region. For the connective tissue, a 60.3%±11.9 mean was observed in the pristine bone; 62.1%±14.5 in the intermediate and 58.5%±13.6 in the apical region. The median for the remaining biomaterial was 1.5 in the pristine bone region; 1 in the intermediate region and 2.5 in the apical region.

The group BO presented lamellar new bone formation in the three regions evaluated in a cellularized connective tissue stroma and much remaining biomaterial. The mean for the new bone formed was 33.4%±12.6 in the pristine bone; 32.5%±10.8 in the intermediate and 34.3%±12.7 in the apical region. The amount of connective tissue formed in the pristine bone region was 38.3%±9.5; in the intermediate region was 40.9%±7.1 and 41.0%±7.2 in the apical region. The median for the remaining biomaterial in this group was 36.0 in pristine bone; 26.5 in intermediate and 25.5 in apical region.

**Figure 2:**
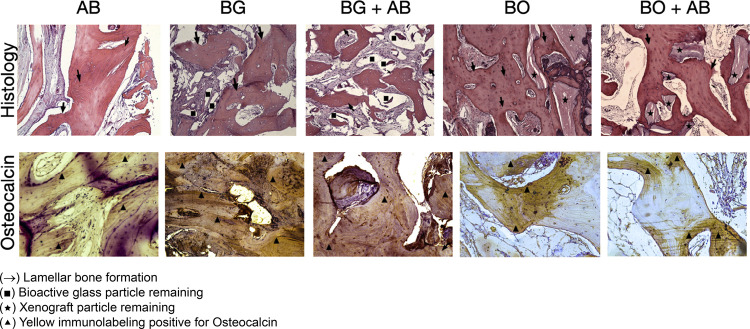
Image showing the histological sections for histology evaluation and immunolabeling positive for Osteocalcin in the groups evaluated

The group BO + AB 1:1 had lamellar bone formation with less remaining biomaterial due to autogenous bone graft particle resorption and less use of xenograft. The mean of the new bone formed in group 5 in the pristine bone, intermediate and apical regions was 32.8%±11.5; 36.1%±16.0 and 27.8%±19.8 respectively. For connective tissue, the mean in the pristine bone region was 46.9%±14.5; in the intermediate region was 40.9%±21.2 and in the apical region was 43.4%±15.9. The remaining biomaterial median was 22.5 for pristine bone; 24.5 for the intermediate and 36.0 for the apical region. Osteoclasts were absent around the bioactive glass or xenografts of specimens. (Figure 2 Histology sections) (Table 1).

**Table 1:** Histometric outcomes for new bone formation, Connective tissue and Biomaterial remaining after 6 months of bone repair in the 5 groups evaluated

Groups	New bone formed			Connective tissue			Biomaterial remaining		
	Pristine bone (%SD)	Intermediate (%SD)	Apical (%SD)	Pristine bone (%SD)	Intermediate (%SD)	Apical (%SD)	Pristine bone (%)	Intermediate (%)	Apical (%)
1	37.8±16.9	38.1±21.4	44.5±18.6	57.6±16.3	58.8±20.9	54.0±16.5	1.5	0.5	2.5
2	43.6±4.7	37.3±10.9	49.3±13.2	56.6±6.5	63.4±11.0	49.3±13.2	0	0	0
3	39.0±15.8	34.8±14.5	36.8±14.5	60.3±11.9	62.1±14.5	58.5±13.6	1.5	1	2.5
4	33.4±12.6	32.5±10.8	34.3±12.7	38.3±9.5	40.9±7.1	41.0±7.2	36.0	26.5	25.5
5	32.8±11.5	36.1±16.0	27.8±19.8	46.9±14.5	40.9±21.9	43.4±15.9	22.5	24.5	36.0

**Figure 3:**
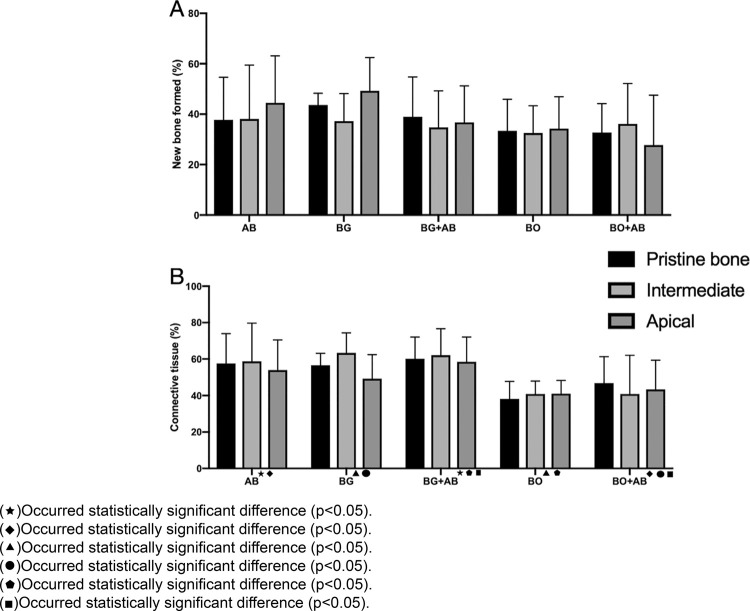
A. Graphic showing the histomorphometric outcomes for new bone formation after 6 months of bone repair in the human maxillary sinus augmented with the 5 bone substitutes studied. B. Graphic showing the histomorphometric outcomes for connective tissue after 6 months of bone repair in the human maxillary sinus augmented with the 5 bone substitutes studied

The difference between new bone formation among groups and regions was not statistically significant (p<0.05). For connective tissue, the difference was statistically significant among groups (p=0.0001). Tukey’s *post hoc* test detected statistically significant differences between groups AB and BG + AB 1:1 (p=0.001); groups AB and BO + AB 1:1 (p=0.012); groups BG and BO (p=0.001); groups BG and BO + AB 1:1 (p=0.023); groups BG + AB 1:1 and BO (p=0.0001) as well as groups BG + AB 1:1 and BO + AB 1:1 (p=0.001). The comparison among groups for remaining biomaterial also had a statistically significant difference (*X*^2^=59.232; p=0.0001). The test showed difference between groups AB and BO (p=0.0001); groups AB and BO + AB 1:1 (p=0.0001); groups BG and BO (p=0.0001); groups BG and BO + AB 1:1 (p=0.0001); groups BG + AB 1:1 and BO (p=0.0001) and groups BG + AB 1:1 and BO + AB 1:1 (p=0.001). Besides this, statistically significant differences were observed among regions for remaining biomaterial by the *post hoc* test (*X*^2^=61.156; p=0.0001): pristine bone region of groups AB and BO (p=0.048), pristine bone of group AB and apical region of group BO (p=0.025); pristine bone region of group AB and apical region of group BO + AB 1:1 (p=0.004). The differences were also observed among the intermediate region of group BG and the pristine bone (p=0.020), intermediate (p=0.022), and apical region (p=0.011) of group BO. The apical region of group BO + AB 1:1 showed difference among pristine bone region of group AB (p=0.004) and the intermediate region (p=0.002) and apical region (p=0.011) of group BG. (Figure 3 A, B)

Therefore, our results were in accordance with hypothesis H_0_.

### Immunohistochemistry evaluation

Group AB presented high level “3” immunolabeling results for osteocalcin, which points to a mature bone that is able to receive dental implants for group BG; for group BG + AB 1:1; for group BO and group BO + AB 1:1 (Figure 2 Osteocalcin immunolabeling sections).

## Discussion

In our study, we evaluated the bone formation in human maxillary sinus bone augmentation using two different bone grafts. Previous literature reports that Biogran and Bio-Oss have different ways of graft resorption. Lindhe, et al.^17^(2010) evaluated Bio-Oss Collagen in human sockets, concluding that Bio-Oss particles were not reabsorbed and bone formation was delayed after 6 months. Hallman, Lundgren and Sennerby^18^ (2001) and Lindgren, et al.^19^ (2012) analyzed bone specimens from human maxillary sinuses augmented with Bio-Oss and found 12.4% and 24.0% of particles after 3 years, respectively. Our study showed a 36.0% median of Bio-Oss in the pristine bone region; 26.5% in the intermediate and 25.5% in the apical region. The presence of osteoclasts around the graft remaining were not observed. Our data are in line with previous studies, which outline that Bio-Oss tends to be “resistant to resorption”.^19^

Biogran has a peculiar way of resorption: body fluids react with these bone substitute due to its unstable structure. A silica gel and calcium phosphate layer are formed around the particle surface, which allow a combined process of chemical dissolution and macrophage action.^10^ With simultaneous colonization of osteoblasts, the particle disintegrates. A rate of bioactive glass particles remaining from 0% to 4.2% was reported after 6 months of bone healing in maxillary sinus bone augmentation, similar to our study, in which we report a 0% median in all regions evaluated.^20^ Thus, we demonstrated that another crack formed after each particle divided, and the occurrence of a new osteoblast activity.

Previous studies show different rates of new bone formation in maxillary sinus bone augmentation using both biomaterials. Rodriguez y Baena et al.^21^ (2017) reported a 27.5% bone formation using Bio-Oss and 16.6% of residual graft. Lee, et al.^22^ (2017) found 26.1% of new bone formed and 25.7% of residual Bio-Oss. Nizam, et al.^23^ (2018) demonstrated a 21.2% bone volume, similar to the outcomes reported in our study. Histometric studies using Biogran alone are scarce. Tadjoedin, et al.^24^ (2002) evaluated it; however, only 3 patients were operated upon and, therefore, their results cannot be considered reliable. Pereira, et al.^20^ (2017) researched the use of Biogran to reconstruct maxillary sinuses compared to autogenous bone graft. Their results showed a 42.0% mean of new bone formation in the pristine bone, 40.7% in the intermediate and 45.6% in the apical region, which is similar to the outcomes of our study.

Despite the autogenous bone graft being the most predictable for bone reconstructions, some authors showed that the requirement of another surgical site leads to post-operative morbidity.^25-27^ Recent studies reported post-surgical complaints immediately after the procedure, and that they tend to decrease in a long term.^13^ Besides this, osteoinductive properties and mesenchymal cells can be transferred with the autogenous bone graft and provide advantages when added to biomaterials to improve the bone quality.^28-30^

In our study, the composite bone graft studied did not result in a statistically significant difference in the rate of new bone formation. Group BG + AB 1:1 showed a mean of 39.0%, 34.8%, and 36.8% in the pristine bone, intermediate region and apical region, respectively. Cordioli, et al.^31^ (2001) reported a 30.6% bone formation rate of Biogran and autogenous bone graft in a 4:1 ratio. Turunen, et al.^32^ (2004) grafted human maxillary sinuses with Biogran and autogenous bone graft from iliac crest in a 1:1 proportion with a 34.0% result, similar to the study of Menezes, et al.^9^(2018).

When Bio-Oss plus autogenous bone graft is used to reconstruct maxillary sinuses, lower rates of new bone is formed. Yildirim, et al.^28^ (2001) showed that 18.9% of bone is formed using this mixture, as well as Bonardi, et al.^8^(2018). When the Bio-Oss associated to A-PRF and i-PRF is used to reconstruct mandible, similar histological findings were identified by Lorenz, et al.^33^ (2018), in which the new bone formed was in direct contact with the biomaterial remaining with lamellar formation. In our study, we also found low rates of new bone formation; however, the histological findings presented lamellar bone and a connective tissue well cellularized. Thus, the H_0_ hypothesis was accepted.

The osteocalcin outcomes demonstrate that all bone substitutes evaluated in this study were calcified and able to receive dental implants, similar to previous studies.^8,15^

The limitation of our study was the evaluation of dental implants in a long-term. According to literature, however, high rates of success are observed when Bio-Oss is used to reconstruct the bone height in maxillary sinuses.^30^ Despite the outcomes showed in this study, further studies are required to evaluate other periods and demonstrate if the biomaterial particle rates influence bone healing.

In conclusion, our study showed the formation of a suitable lamellar bone in the patients that underwent maxillary sinus reconstruction after six months of bone healing in all groups, thus being indicated to receive dental implants.
